# Dpr Acts as a Molecular Switch, Inhibiting Wnt Signaling when Unphosphorylated, but Promoting Wnt Signaling when Phosphorylated by Casein Kinase Iδ/ε

**DOI:** 10.1371/journal.pone.0005522

**Published:** 2009-05-15

**Authors:** Evelyn Teran, Aron D. Branscomb, Joni M. Seeling

**Affiliations:** Department of Biology, City University of New York, Queens College, Flushing, New York, United States of America; University of Washington, United States of America

## Abstract

The Wnt pathway is a key regulator of development and tumorigenesis. Dpr (Dact/Frodo) influences Wnt signaling in part through the interaction of its PDZ-B domain with Dsh's PDZ domain. Studies have shown that XDpr1a and its close relative, Frodo, are involved in multiple steps of the Wnt pathway in either inhibitory or activating roles. We found that XDpr1a is phosphorylated by casein kinase Iδ/ε (CKIδ/ε), an activator of Wnt signaling, in the presence of XDsh. Abrogating XDpr1a's ability to bind XDsh through mutation of XDpr1a's PDZ-B domain blocks CK1δ/ε's phosphorylation of XDpr1a. Conversely, XDsh possessing a mutation in its PDZ domain that is unable to bind XDpr1a does not promote XDpr1a phosphorylation. Phosphorylation of XDpr1a and XDsh by CKIδ/ε decreases their interaction. Moreover, the phosphorylation of XDpr1a by CKIδ/ε not only abrogates XDpr1a's promotion of β-catenin degradation but blocks β-catenin degradation. Our data suggest that XDpr1a phosphorylation by CKIδ/ε is dependent on the interaction of XDpr1a's PDZ-B domain with XDsh's PDZ domain, and that the phosphorylation state of XDpr1a determines whether it inhibits or activates Wnt signaling.

## Introduction

In canonical Wnt signaling, Wnt activates β-catenin-dependent transcription via a phosphorylation-regulated signal transduction cascade. In the absence of Wnt, the cytoplasmic β-catenin degradation complex, whose core components include β-catenin, adenomatous polyposis coli (APC), axin, and glycogen synthase kinase 3β (GSK3β), is stable and casein kinase Iα (CKIα) primes β-catenin by phosphorylating Ser45, which is required for GSK3β to phosphorylate three upstream Ser/Thr residues [1]. Phosphorylated β-catenin is then ubiquitinated and degraded by the proteasome. When Wnt binds to low density lipoprotein receptor-related protein 5/6 (LRP5/6) and frizzled (fz) coreceptors, an intracellular signaling cascade is activated that includes axin binding to LRP5/6, and disheveled (Dsh/Dvl) binding to fz, bringing β-catenin and APC to the membrane as well [2]–[6]. CKIε positively regulates Wnt signaling, potentially by phosphorylating Dsh, and LRP5/6 is phosphorylated by GSK3β and CKI [7]–[9]. These events inactivate the β-catenin degradation complex, reduce β-catenin phosphorylation, and increase β-catenin abundance. β-catenin then forms a complex with a Lef/Tcf transcription factor, activating transcription of dorsalizing factors in early *Xenopus* development and cell cycle regulators in mammalian cells [10]–[12].

Dsh has three highly conserved domains: an N-terminal DIX domain (which is also present in axin), a central PDZ domain (first identified in PSD, Discs large, and ZO1), and a C-terminal DEP domain (first identified in Dsh, Egl-10, and Pleckstrin) [13], [14]. Dsh's DIX and PDZ domains are likely to function in canonical Wnt signaling, while its PDZ and DEP domains are likely to function in noncanonical Wnt signaling [15]–[17]. Dsh is thought to act as a molecular scaffold in signal transduction processes, since at least eighteen binding partners of Dsh have been identified [14]. PDZ domains are protein–protein interaction domains and therefore are important for the scaffolding function of Dsh. *Xenopus* Dapper1a (XDpr1a) and the highly related protein Functional regulator of disheveled in ontogenesis (Frodo), are novel mediators of Wnt signaling which were isolated in independent yeast two-hybrid screens for proteins that interact with *Xenopus* Dsh (XDsh) [18], [19]. XDpr1a and Frodo have two defined motifs: an N-terminal leucine zipper (LZ) domain, and a C-terminal PDZ-binding (PDZ-B) domain [18], [19]. The LZ domain is unnecessary for XDpr1a's interaction with XDsh, since deletion of the LZ domain does not influence XDpr1a's ability to associate with XDsh, although it does affect the ability of XDpr1a to inhibit Wnt signaling [18]. However, deletion or mutation of the PDZ-B domain impedes XDpr1a or Frodo from associating with XDsh [18], [19]. Recently, a domain was identified in the central region of human Dpr1 that also mediates Dvl binding, but the role of this region in XDpr1a is not known [20].

XDpr1a and Frodo are the result of a recent gene duplication in *Xenopus* and intriguingly, studies have shown that XDpr1a and Frodo are involved in multiple steps of the Wnt pathway in either inhibitory or activating roles [21], [22]. In many instances, XDpr1a and/or Frodo negatively regulate Wnt signaling. XDpr1a forms a complex with XDsh, axin, GSK3β, CKIε, and β-catenin; exogenous XDpr1a increases axin and GSK3β, and reduces CKIε, in this complex, resulting in reduced β-catenin abundance and reduced activation of Wnt responsive genes [18]. Furthermore, Dpr inhibits Wnt signaling both from the cytoplasm and the nucleus. Mammalian Dpr1/Dact inhibits expression of Wnt-responsive reporters through its promotion of Dvl degradation in a lysosome-dependent pathway, and by inhibiting the binding of LEF1 with β-catenin, but promoting the binding of LEF1 with a corepressor, histone deacetylase 1 (HDAC1) [20], [23].

In contrast, XDpr1a and/or Frodo also activate Wnt signaling. Reducing Frodo abundance with antisense morpholino oligonucleotides (MOs) inhibits Xwnt8- and XDsh-induced body axes and reduces Wnt-dependent reporter activity in *Xenopus* [18], [19]. In addition, Dpr1 enhances Wnt8's ventralization and posteriorization activities in zebrafish [21], [22]. Using a similar experimental system, however, Frodo and XDpr1a were found to either inhibit or activate Wnt signaling, dependent on the point at which the pathway was activated [22]. Frodo and XDpr1a MOs reduce β-catenin-independent transcription induced by TCF-VP16, suggesting that Frodo and XDpr1a are required for Tcf-mediated transcription, and Frodo and XDpr1a MOs increase Xwnt8-induced siamois reporter activity, suggesting that Frodo and XDpr1a inhibit Wnt signaling. The molecular mechanisms underlying these apparent differences in function have not yet been explored.

Phosphorylation plays a key role in the regulation of Wnt signaling, and CKI family members appear to have several targets in the Wnt pathway. CKIα phosphorylates β-catenin to negatively regulate the pathway [24]. CKIδ and CKIε are two highly related CKI isoforms likely to have similar functions, since their kinase domains are 98% identical and they have 53% identical C-terminal tails, unique to CKIδ and CKIε, that inhibit their function when autophosphorylated. CKIδ and CKIε activate Wnt signaling through their putative targets of Dsh and/or Lef/Tcf, while CKIγ activates Wnt signaling through LRP5/6 phosphorylation [24]. We have shown that CKIδ and CKIε both interact directly with Dvl-1, suggesting that they influence Wnt signaling using a common mechanism [8]. CKIδ directly phosphorylates numerous components of the β-catenin degradation complex *in vitro*, i.e., Dsh, APC, axin, and β-catenin, and CKIε phosphorylates axin and β-catenin *in vivo* [8]. In addition, we found that CKIε dissociates protein phosphatase 2A (PP2A) A and C subunits from the β-catenin degradation complex both *in vitro* and *in vivo*, and that CKIδ and CKIε both act upstream of the B56α regulatory subunit of PP2A in *Xenopus* body axis formation, which is a Wnt-dependent process [8].

The circumstances that determine whether Dpr inhibits or activates Wnt signaling are not known. An appealing hypothesis is that posttranslational modifications of Dpr may regulate its role in Wnt signaling. Since phosphorylation is a common mechanism to alter a protein's activity, we analyzed the phosphorylation of Dpr and found that XDpr1a is phosphorylated by CKIδ/ε in the presence of XDsh. CKIδ's phosphorylation of XDpr1a reduces its interaction with XDsh and transforms XDpr1a from a promoter to an inhibitor of β-catenin degradation. Our data suggest that XDpr1a inhibits Wnt signaling when unphosphorylated and bound to XDsh, but activates Wnt signaling when phosphorylated by CKIδ/ε and associated with other Wnt players.

## Results

### XDsh promotes a CKIδ-induced phosphorylation of XDpr1a

**Figure 1:**
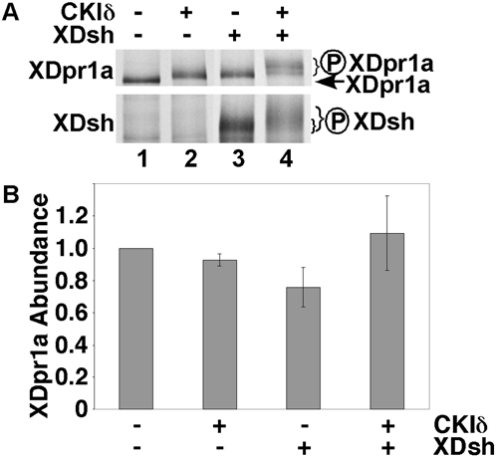
XDsh promotes a CKIδ-mediated mobility shift of XDpr1a. A. XDpr1a exhibits a mobility shift in the presence of CKIδ and XDsh. *In vitro* transcribed and translated XDpr1a exhibits a mobility shift in the presence of purified CKIδ, and in the presence of *in vitro* transcribed and translated XDsh. The mobility shift is greater in the presence of both CKIδ and XDsh. XDsh also exhibits a mobility shift in the presence of CKIδ. The XDpr1a mobility shift present in lanes 2 and 3 is likely due to limiting amounts of endogenous XDsh and CKIδ in the reticulocyte lysates used in the *in vitro* transcription and translation, respectively. B. XDsh-mediated CKIδ phosphorylation of XDpr1a has little effect on XDpr1a abundance. Phosphorylation reactions were carried out as in A., but with the inclusion of luciferase as a loading control. XDpr1a and luciferase bands were quantitated, and the XDpr1a signal was normalized to that of luciferase. The luciferase-normalized signals were then normalized to that of XDpr1a alone. Error bars signify standard deviation (n = 3 trials).

To study the phosphorylation of XDpr1a, we first examined the ability of two Wnt pathway kinases to phosphorylate XDpr1a *in vitro*, GSK3β and CKIδ/ε. We found that CKIδ, but not GSK3β, was able to phosphorylate XDpr1a, as exemplified by a gel shift (Fig. 1A, compare lane 2 with lane 1, and data not shown). XDpr1a was initially isolated because of its interaction with XDsh, and XDsh is phosphorylated by CKIδ/ε *in vitro* [8], [18], [19], so we determined if XDsh influences the phosphorylation state of XDpr1a. We found that XDsh induced an upward mobility shift of XDpr1a similar to the shift seen in the presence of CKIδ (Fig. 1A, compare lane 3 with lane 1). Note that XDpr1a migrates slower than its calculated molecular weight of 91 kD, likely due to as yet uncharacterized posttranslational modifications. Intriguingly, the presence of both CKIδ and XDsh resulted in a hypershift of XDpr1a (Fig. 1A, compare lane 4 with lanes 1–3), suggesting that XDsh promotes the phosphorylation of XDpr1a by CKIδ. This also suggests that the modest gel shift seen in the presence of CKIδ alone (Fig. 1A, lane 2) was due to limiting amounts of endogenous Dsh in reticulocyte lysates, whereas the modest gel shift in the presence of XDsh alone (Fig. 1A, lane 3) was due to limiting amounts of endogenous CKIδ in reticulocyte lysates, and that the supershift in the presence of XDsh and CKIδ (Fig. 1A, lane 4) occurs when neither XDsh nor CKIδ are limiting. As reported previously [8], [18], [19], we found that CKIδ also phosphorylates XDsh (Fig. 1, compare lane 4 with lane 3). The broad XDsh band in the absence of CKIδ (Fig. 1A, lane 3) suggests that endogenous CKIδ and/or other kinases in the reticulocyte lysate phosphorylate XDsh.

**Figure 2:**
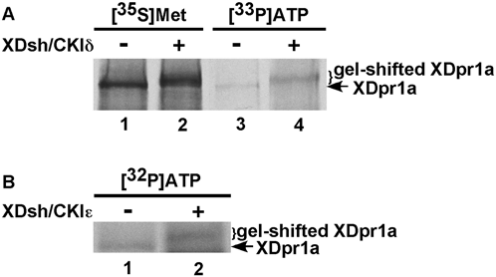
XDsh promotes the phosphorylation of XDpr1a by CKIδ both *in vitro* and *in vivo*. A. XDsh induces a CKIδ-mediated phosphorylation of XDpr1a *in vitro*. Phosphorylation reactions were carried out in the presence of [^35^S]methionine-XDpr1a (lanes 1 and 2) or [γ-^33^P]ATP (lanes 3 and 4) and in the absence (lanes 1 and 3) or presence (lanes 2 and 4) of XDsh and CKIδ. Lanes 3 and 4 contain the immunopellet from an anti-Myc immunoprecipiation of [γ-^33^P]ATP-labeled Myc:XDpr1a. XDpr1a undergoes a gel-shift and shows increased incorporation of [γ-^33^P]ATP in the presence of XDsh and CKIδ. B. CKIε phosphorylates XDpr1a *in vivo*. HEK293 cells transfected with Flag:XDpr1a alone or with CKIε and XDsh were metabolically labeled with [^32^P]orthophosphoric acid prior to XDpr1a immunoprecipitation with anti-Flag antibodies. The cotransfection of CKIε and XDsh with XDpr1a induces a gel-shift and increases [^32^P]orthophosphoric acid incorporation into XDpr1a. This result is representative of experiments repeated three times with similar results.

Because mobility shifts of phosphorylated proteins can cause the broadening of SDS-PAGE bands and make it difficult to estimate protein abundance determinations by eye, we quantitated the XDpr1a signal from CKIδ phosphorylation reactions to determine if CKIδ-mediated phosphorylation of XDpr1a affects XDpr1a abundance. We found that the abundance of XDpr1a is relatively constant in the presence of XDsh and/or CKIδ (Fig. 1B). This suggests that while CKIδ phosphorylates XDpr1a, it has little effect on XDpr1a abundance. To verify that the XDsh/CKIδ-mediated XDpr1a gel shift was due to phosphorylation, we carried out CKIδ phosphorylation reactions in the presence of [γ-^33^P]ATP. The presence of XDsh and CKIδ induced incorporation of [γ-^33^P]ATP into XDpr1a (Fig. 2A, compare lane 4 to 3), which indicates XDpr1a is phosphorylated under these conditions, and induced a gel-shift that comigrates with the [^35^S]methionine-labeled XDpr1a gel-shift (Fig. 2A, compare lane 4 to 3 and lane 2 to 1), showing that the gel-shifted XDpr1a is phosphorylated. The gel-shift of XDpr1a represents a 4.0±0.7% increase in molecular weight, or approximately 6 kD. The data confirm that the XDsh/CKIδ-mediated gel-shift of XDpr1a is due to XDpr1a phosphorylation. In summary, XDsh promotes the phosphorylation of XDpr1a by CKIδ, perhaps by bridging XDpr1a to CKIδ.

### CKIε phosphorylates XDpr1a *in vivo*

To examine the phosphorylation of XDpr1a by CKIδ/ε under more physiological conditions, we monitored the effects of modulating CKIδ/ε activity on XDpr1a phosphorylation *in vivo*. HEK293 cells were transfected with Myc:XDpr1a alone or with HA:XDsh and CKIε, and metabolically labeled with [^32^P]orthophosphoric acid. CKIε induced an XDpr1a gel-shift and promoted increased incorporation of [^32^P]orthophosphoric acid into XDpr1a (Fig. 2B). The gel-shift of XDpr1a represents a 9.1±0.8% increase in molecular weight, or approximately 13 kD, somewhat higher than the *in vitro* shift of 6 kD, suggesting that XDpr1a phosphorylation is more robust *in vivo*. This result extends our *in vitro* data and shows that XDpr1a is phosphorylated by CKIε *in vivo*.

### PDZ-B mutants of XDpr1a are not phosphorylated by CKIδ

**Figure 3:**
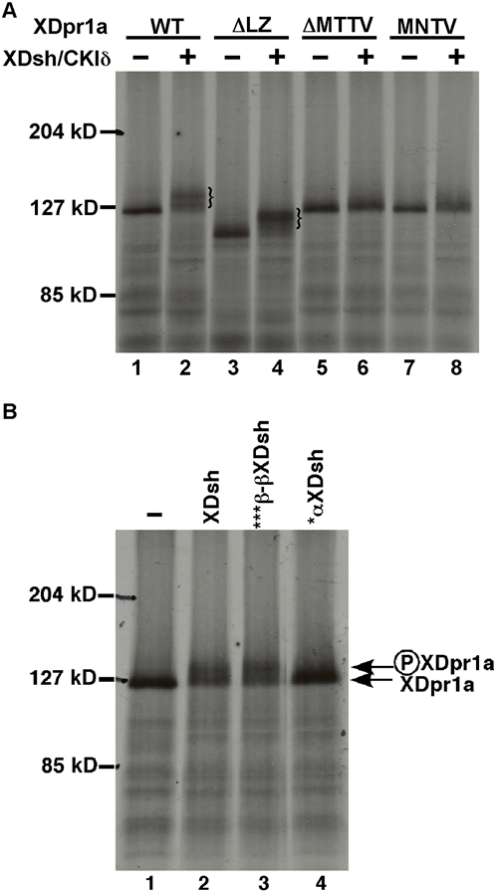
Mutations of XDpr1a or XDsh that block their mutual interaction also block CKIδ-mediated XDpr1 phosphorylation. A. Deletion or mutation of XDpr1a's PDZ-B domain blocks CKIδ-mediated XDpr1a phosphorylation. Deletion of the leucine zipper domain of XDpr1a (ΔLZ), which does not affect its ability to bind XDsh, does not affect the ability of XDpr1a to be phosphorylated by CKIδ, as exhibited by a mobility shift. XDpr1a containing a deletion (ΔMTTV) or a point mutation (MNTV) of its PDZ-B domain is not phosphorylated by CKIδ. The braces in lanes 2 and 4 bracket phosphorylated XDpr1a and ΔLZ, respectively. B. An Asn317Thr Mutation in XDsh's PDZ domain abrogates its promotion of XDpr1a phosphorylation. ***β-βXDsh, which contains Gln272Ala, Ser273Ala, and Glu275Ala mutations in a PDZ domain loop outside of the PDZ-B binding domain, promotes XDpr1a phosphorylation by CKIδ at a level similar to that of wild-type XDsh, while *αXDsh, which contains an Asn317Thr mutation in the PDZ-B binding domain within its PDZ domain, does not.

Mutational analyses have shown that the PDZ-B domain of XDpr1a/Frodo interacts with the PDZ domain of XDsh [18], [19]. If XDsh is required to promote the phosphorylation of XDpr1a by CKIδ, then mutants of XDpr1a with reduced XDsh binding may not be phosphorylated by CKIδ. We tested this hypothesis by determining if mutation or deletion of the XDpr1a PDZ-B domain abrogated the ability of XDsh to promote CKIδ-mediated XDpr1a phosphorylation. We used *in vitro* transcription/translation to synthesize wild-type and mutant XDpr1a proteins, as well as XDsh, followed by a phosphorylation reaction in the presence of purified CKIδ. We tested three XDpr1a mutants, one that binds XDsh's PDZ domain (XDpr1aΔLZ), and two that do not (XDpr1aΔMTTV and XDpr1aM**N**TV) [18]. The XDpr1aΔLZ protein, lacking the N-terminal 129 amino acids including the leucine zipper motif, served as a control and behaved similarly to wild-type XDpr1a, undergoing a gel shift indicative of hyperphosphorylation in the presence of CKIδ (Fig. 3A, compare lane 2 with lane 1, and lane 4 with lane 3). XDpr1aΔMTTV lacks the PDZ-B domain and did not undergo a mobility shift in the presence of CKIδ, suggesting that it is not phosphorylated by CKIδ (Fig. 3A, compare lane 6 with lane 5). Since deletion of the XDpr1a PDZ-B domain inhibited XDsh's promotion of XDpr1a phosphorylation by CKIδ, we examined whether a point mutation within the PDZ-B motif would affect XDsh-dependent CKIδ phosphorylation of XDpr1a. We found that XDpr1aM**N**TV, containing a T822N point mutation in its PDZ-B domain, behaved similarly to XDpr1aΔMTTV, and did not exhibit a mobility shift (Fig. 3A, compare lane 8 with lane 7). These data suggest that an intact PDZ-B domain in XDpr1a is required for XDsh-dependent phosphorylation of XDpr1a by CKIδ.

### Mutation of the XDsh PDZ-B binding domain blocks XDpr1a phosphorylation

To further show the importance of XDsh in the phosphorylation of XDprIa by CKIδ, we examined whether a mutation in XDsh that reduces its interaction with XDpr1a's PDZ-B domain still promotes the phosphorylation of XDpr1a by CKIδ. The region of XDsh's PDZ domain that binds to XDpr1a's PDZ-B was identified by X-ray crystallography [18]. An N317T point mutation in XDsh's PDZ-B binding domain (*αXDsh) diminishes its interaction with XDpr1a, whereas a triple mutation in a PDZ domain loop upstream of XDsh's PDZ-B binding domain (272**QS**N**E**275 to 272**AA**N**A**275**,** ***β-βXDsh) does not [18]. We examined the ability of *αXDsh and ***β-βXDsh to promote the phosphorylation of XDpr1a by CKIδ. XDpr1a exhibited a mobility shift indicative of hyperphosphorylation in the presence of XDsh and ***β-βXDsh (Fig. 3B, compare lanes 2 and 3 to lane 1), whereas XDpr1a did not exhibit a mobility shift in the presence of *αXDsh (Fig. 3B, lane 4 compared to lane 1). The inability of an XDsh protein containing a point mutation in its PDZ-B binding domain to promote the phosphorylation of XDpr1a by CKIδ suggests that XDsh must retain its ability to bind XDpr1a in order to promote XDpr1a phosphorylation.

### CKIδ reduces the interaction between XDpr1a and XDsh

**Figure 4:**
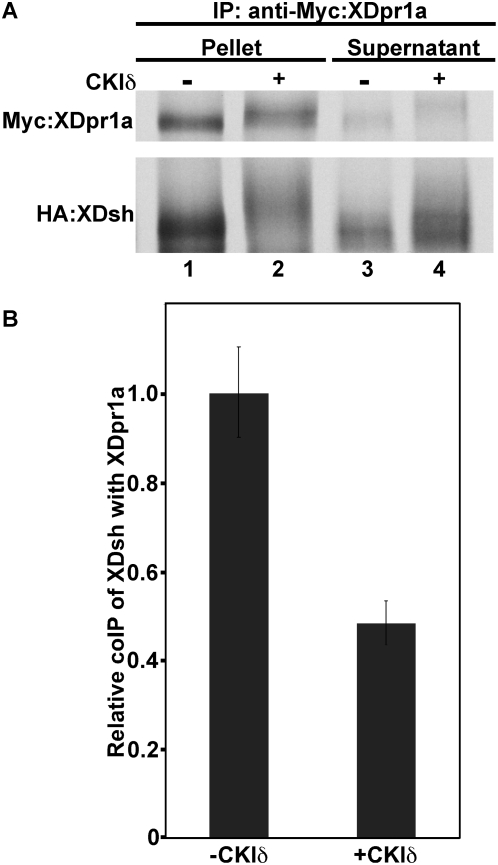
Phosphorylation of XDpr1a and XDsh by CKIδ reduces their interaction. A. Myc-tagged XDpr1a was immunoprecipitated in the presence of HA-tagged XDsh in the absence or presence of CKIδ. The presence of CKIδ reduced the coimmunoprecipitation of XDsh with XDpr1a. B. Quantitation of the relative coimmunoprecipitation (coIP) of XDsh with XDpr. The quantitation of the coimmunoprecipitation of XDsh with XDpr1a revealed that the presence of CKIδ reduced the interaction between XDpr1a and XDsh by approximately one-half when compared to the control. Error bars signify standard deviation.

CKIδ/ε destabilizes the β-catenin degradation complex [8], and Dpr and Dsh are both components of this complex, so CKIδ may disrupt the interaction between Dpr and Dsh as well. We tested this hypothesis using an *in vitro* coimmunoprecipitation assay. We immunoprecipitated Myc-tagged XDpr1a from a reaction containing HA-tagged XDsh in the absence or presence of CKIδ. The presence of CKIδ dramatically decreased the immunoprecipitation of XDsh with XDpr1a (Fig. 4A, compare lanes 2 and 1), resulting in a concomitant increase of XDsh in the immunosupernatant (Fig. 4A, compare lanes 4 and 3). Because of the CKIδ-induced mobility shift of XDsh, determination of the extent of the reduction of XDsh coimmunoprecipitation in the presence of CKIδ is difficult to make by eye, therefore we quantitated the coimmunoprecipitatipon of XDsh. We found that CKIδ reduced the coimmunoprecipitation of XDsh by XDpr1a by approximately one-half (Fig. 4B). This suggests that XDpr1a and XDsh interact transiently, dissociating soon after XDpr1a and/or XDsh is phosphorylated by CKIδ. Therefore, CKIδ reduces the interaction between XDsh and XDpr1a and is likely to play a significant role in Wnt signaling activation.

### XDpr1a promotes β-catenin degradation in the absence of CKIδ, but inhibits β-catenin degradation in its presence

**Figure 5:**
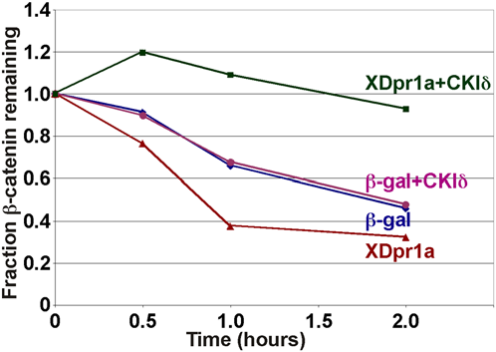
Unphosphorylated XDpr1a promotes, but CKIδ-phosphorylated XDpr1a blocks, β-catenin degradation. Myc:XDpr1a was added to an *in vitro* β-catenin degradation assay after preincubation with or without CKIδ followed by anti-Myc immunoprecipitation. β-galactosidase preincubated with or without CKIδ was used as a control. Untreated XDpr1a promoted β-catenin degradation, whereas XDpr1a preincubated with CKIδ blocked β-catenin degradation. The data shown represent assays repeated six times.

XDpr1a has been shown to reduce β-catenin abundance both in *Xenopus* embryos and in mammalian tissue culture [18]. To investigate the functional consequences of XDsh-mediated CKIδ phosphorylation of XDpr1a, we utilized an *in vitro* β-catenin degradation assay using *Xenopus* egg extracts [8], [25], [26]. When unmodified XDpr1a was added to *Xenopus* egg extracts, the rate of β-catenin degradation increased, reducing the half-life of β-catenin approximately two-fold, from 1.8 to 0.9 hours, indicating an inhibition of canonical Wnt signaling (Fig. 5). However, when XDpr1a was preincubated with CKIδ, β-catenin degradation was blocked, indicative of Wnt pathway activation (Fig. 5). These data suggest that XDpr1a acts as a molecular switch, inhibiting Wnt signaling when unphosphorylated, but promoting Wnt signaling when phosphorylated by CKIδ.

## Discussion

XDpr1a is a member of a conserved family of novel Dsh binding proteins. XDpr1a's PDZ-B domain interacts with XDsh's PDZ domain [18]. Here, we show that XDsh mediates the phosphorylation of XDpr1a by CKIδ. In addition, we found that an intact PDZ-B domain in XDpr1a, as well as an intact PDZ-B binding domain in XDsh, is required for XDsh-dependent phosphorylation of XDpr1a by CKIδ. This suggests that XDpr1a and CKIδ/ε do not interact directly and/or robustly with one another, and that XDsh is required to link XDpr1a and CKIδ/ε.

Epigenetic regulation of Dpr expression is associated with tumorigenesis. Human Dpr1/DACT1 is often downregulated by allelic loss or promoter methylation in hepatocellular carcinomas [27], [28]. There is reduced DACT3 expression in human colon tumors due to histone modifications, resulting in increased Wnt signaling activity; this suppression of DACT3 expression is relieved in colon cancer cell lines by treatment with histone methylation and deacetylase inhibitors [28], [29]. In addition, Dpr is upregulated by the treatment of breast cancer patients with DNA methylation and histone deacetylase inhibitors [28], [29]. Each of these results suggests that the dominant function of Dpr with regard to tumor formation is inhibition of Wnt signaling, and that the loss of this function is associated with tumorigenesis. Our data show that XDpr1a is phosphorylated by CKIδ/ε, and that this phosphorylation reduces XDpr1a's interaction with XDsh. Frizzled-1 overexpression, which activates Wnt signaling, results in disparate localizations of XDsh and XDpr1a, causing the membrane localization of XDsh but not XDpr1a [18]. CKIδ's ability to reduce XDsh/XDpr1a binding may play a part in Wnt's localization of XDsh and XDpr1a to disparate locations. Intriguingly, we found that XDpr1a promotes β-catenin degradation when unphosphorylated but blocks β-catenin degradation when phosphorylated by CKIδ. Overall, our data suggest that XDpr1as acts as a molecular switch in Wnt signaling. In the absence of CKIδ/ε activity, XDpr1a is bound to XDsh and inhibits Wnt signaling, whereas XDpr1a promotes Wnt signaling when phosphorylated by CKIδ/ε.

Cong et al proposed that the N-terminal region of Dsh, containing the DIX domain, is required for its canonical Wnt signaling activity, and that Dsh's C-terminal region, containing the PDZ and DEP domains, structurally blocks this function [30]. In addition, they suggest that this inhibition is relieved by the phosphorylation of Dsh by CKIε. Our data suggest that Dpr is a missing link in the inhibition of Dsh function. We propose that Dpr inhibits Dsh's function in canonical Wnt signaling by binding to Dsh's PDZ domain, and preventing Dsh's DIX domain from activating Wnt signaling. Further, the phosphorylation of XDpr1a and XDsh by CKIδ/ε relieves this suppression by phosphorylating both XDpr1a and XDsh and reducing their interaction.

We propose a model for the regulation of Wnt signaling based on our data, as well as that from several other labs, in which the phosphorylation state of Dpr acts as a molecular switch to determine whether Dpr inhibits or activates Wnt signaling. In the absence of Wnt, unphosphorylated XDpr1a binds to XDsh in the β-catenin degradation complex and inhibits Wnt signaling. When Wnt binds to LRP5/6 and fz coreceptors, the β-catenin degradation complex moves to the membrane through the interactions of axin with LRP5/6 and Dsh with fz, [2]–[6]. This results in CKIδ/ε-mediated phosphorylation of XDsh, XDpr1a, and other substrates [24]. The β-catenin degradation complex then partially dissociates, with β-catenin, APC, and XDsh remaining at the membrane, while PP2A A and C subunits [8] and XDpr1a are released from the complex. Phosphorylated XDpr1a then associates with distinct Wnt pathway binding partners and activates Wnt signaling. Future experimentation will determine if the phosphorylation state of XDpr1a affects its association with other known Wnt pathway protein partners. Our data suggest that LEF1, HDAC, and β-catenin may differentially associate with unphosphorylated XDpr1a, while Tcf3 may differentially associate with CKIδ/ε-phosphorylated XDpr1a.

## Materials and Methods

### *In vitro* phosphorylation assay

*In vitro* transcription and translation was performed with [^35^S]methionine (Amersham Biosciences Corp., Piscataway, NJ) using TNT SP6 Quick Coupled Transcription/Translation System (Promega, Madison, WI) according to the manufacturer's instructions. Unlabeled proteins were prepared with TNT SP6 Quick Coupled Transcription/Translation System using cold methionine. XDpr1a and XDsh TNT reactions were mixed at a ratio of 1:1 in a reaction also containing 5 mM MgCl_2_ and 0.5 mM ATP. Rat CKIδ lacking its autoinhibitory C-terminal domain (New England Biolabs, Ipswich, MA) or GSK3β (New England Biolabs, Ipswich, MA), was added to a final concentration of 1.1 µM, and the phosphorylation reactions were carried out for 1.5 hours at 30°C. Where specified, [^35^S]methionine-labeled luciferase was added to the phosphorylation reactions, and bands were quantitated after denaturing SDS-PAGE using a Molecular Dynamics PhosphorImager and ImageQuant software. Where specified, [γ-^33^P]ATP was added to the CKIδ reactions followed by a 1.5 hour incubation at 30°C.

### Cell culture

For *in vivo* labeling, HEK293 cells were transfected with Flag:XDpr1a, HA:XDsh, and CKIε, or Flag:XDpr1a with empty vector, using Lipofectamine Plus (Invitrogen, Carlsbad, CA) and metabolically labeled with [^32^P]orthophosphoric acid (PerkinElmer, Boston, MA). Cells were homogenized in lysis buffer (50 mM Tris 7.5, 150 mM NaCl, 1% Triton X-100, 100 µM NaF, 0.5 mM Na_3_VO_4_, 10 mM β-glycerol phosphate), followed by anti-Flag immunoprecipitations, SDS-PAGE, and visualization using a Molecular Dynamics PhosphorImager. XDpr1a's molecular weight was determined in the absence or presence of CKIε and XDsh from three experimental trials using GelScape (www.gelscape.ualberta.ca:8080/htm/index.html).

### *In vitro* coimmunoprecipitations

[γ-^33^P]ATP-labeled Myc:XDpr1a was immunoprecipitated in the presence of anti-Myc beads for 2 hours at room temperature. Immunoprecipitates were washed three times with 50 mM Tris-HCl pH 7.4, 137 mM NaCl, followed by SDS-PAGE and visualization by autoradiography. XDpr1a's molecular weight was determined in the absence or presence of CKIδ and XDsh from three experimental trials using GelScape (www.gelscape.ualberta.ca:8080/htm/index.html). [^35^S]methionine-labeled Myc:XDpr1a was immunoprecipitated in the presence of anti-Myc antibodies and protein A agarose at room temperature for 2 hours. Immunoprecipitates were washed three times with 50 mM Tris-HCl pH 7.4, 137 mM NaCl, followed by denaturing SDS-PAGE, visualization by autoradiography, and quantitation using a Molecular Dynamics PhosphorImager and ImageQuant software. The charted coimmunoprecipitation data is the percentage of XDsh in the immunoprecipitate (versus total input), divided by the percentage of XDpr1a in the immunoprecipitate (versus total input), normalized to 1.0 in the absence of CKIδ (*n* = 4 trials).

### β-catenin degradation assays

*Xenopus* egg extracts were prepared, RNA was synthesized and translated, and degradation assays were carried out as described previously with minor modifications [8], [25], [26]. Myc:XDpr1a or β-galactosidase was preincubated with or without CKIδ following its translation in egg extracts. Anti-Myc immunoprecipitates were washed prior to being added to fresh egg extract for the degradation assay, which contained 40 µM IC261 to inhibit any potential carryover CKIδ activity. [^35^S]β-catenin was synthesized using TNT T7 coupled wheat germ extract system (Promega, Madison, WI). Degradation assays were performed six times, with aliquots removed at 0, 0.5, 1.0, and 2.0 hours. Aliquots were resolved using SDS-PAGE, imaged using a Molecular Dynamics PhosphorImager, and quantitated using ImageQuant software.

## References

[1] Liu C, Li Y, Semenov M, Han C, Baeg GH (2002). Control of beta-catenin phosphorylation/degradation by a dual-kinase mechanism.. Cell.

[2] He X, Semenov M, Tamai K, Zeng X (2004). LDL receptor-related proteins 5 and 6 in Wnt/beta-catenin signaling: arrows point the way.. Development.

[3] Yanagawa S, van Leeuwen F, Wodarz A, Klingensmith J, Nusse R (1995). The dishevelled protein is modified by wingless signaling in Drosophila.. Genes Dev.

[4] Wong HC, Bourdelas A, Krauss A, Lee HJ, Shao Y (2003). Direct binding of the PDZ domain of Dishevelled to a conserved internal sequence in the C-terminal region of Frizzled.. Mol Cell.

[5] Schwarz-Romond T, Metcalfe C, Bienz M (2007). Dynamic recruitment of axin by Dishevelled protein assemblies.. J Cell Sci.

[6] Hendriksen J, Jansen M, Brown CM, van der Velde H, van Ham M (2008). Plasma membrane recruitment of dephosphorylated beta-catenin upon activation of the Wnt pathway.. J Cell Sci.

[7] Zhang L, Jia J, Wang B, Amanai K, Wharton KA (2006). Regulation of wingless signaling by the CKI family in Drosophila limb development.. Dev Biol.

[8] Gao ZH, Seeling JM, Hill V, Yochum A, Virshup DM (2002). Casein kinase I phosphorylates and destabilizes the beta-catenin degradation complex.. Proc Natl Acad Sci U S A.

[9] Zeng X, Tamai K, Doble B, Li S, Huang H (2005). A dual-kinase mechanism for Wnt co-receptor phosphorylation and activation.. Nature.

[10] He TC, Sparks AB, Rago C, Hermeking H, Zawel L (1998). Identification of c-MYC as a target of the APC pathway.. Science.

[11] Mann B, Gelos M, Siedow A, Hanski ML, Gratchev A (1999). Target genes of beta-catenin-T cell-factor/lymphoid-enhancer-factor signaling in human colorectal carcinomas.. Proc Natl Acad Sci U S A.

[12] Tetsu O, McCormick F (1999). Beta-catenin regulates expression of cyclin D1 in colon carcinoma cells.. Nature.

[13] Boutros M, Mlodzik M (1999). Dishevelled: at the crossroads of divergent intracellular signaling pathways.. Mech Dev.

[14] Wharton KA (2003). Runnin' with the Dvl: proteins that associate with Dsh/Dvl and their significance to Wnt signal transduction.. Dev Biol.

[15] Axelrod JD, Miller JR, Shulman JM, Moon RT, Perrimon N (1998). Differential recruitment of Dishevelled provides signaling specificity in the planar cell polarity and Wingless signaling pathways.. Genes Dev.

[16] Boutros M, Paricio N, Strutt DI, Mlodzik M (1998). Dishevelled activates JNK and discriminates between JNK pathways in planar polarity and wingless signaling.. Cell.

[17] Moriguchi T, Kawachi K, Kamakura S, Masuyama N, Yamanaka H (1999). Distinct domains of mouse dishevelled are responsible for the c-Jun N-terminal kinase/stress-activated protein kinase activation and the axis formation in vertebrates.. J Biol Chem.

[18] Cheyette BN, Waxman JS, Miller JR, Takemaru K, Sheldahl LC (2002). Dapper, a Dishevelled-associated antagonist of beta-catenin and JNK signaling, is required for notochord formation.. Dev Cell.

[19] Gloy J, Hikasa H, Sokol SY (2002). Frodo interacts with Dishevelled to transduce Wnt signals.. Nat Cell Biol.

[20] Zhang L, Gao X, Wen J, Ning Y, Chen YG (2006). Dapper 1 antagonizes Wnt signaling by promoting dishevelled degradation.. J Biol Chem.

[21] Waxman JS, Hocking AM, Stoick CL, Moon RT (2004). Zebrafish Dapper1 and Dapper2 play distinct roles in Wnt-mediated developmental processes.. Development.

[22] Hikasa H, Sokol SY (2004). The involvement of Frodo in TCF-dependent signaling and neural tissue development.. Development.

[23] Gao X, Wen J, Zhang L, Li X, Ning Y (2008). Dapper1 is a nucleocytoplasmic shuttling protein that negatively modulates Wnt signaling in the nucleus.. J Biol Chem.

[24] Price MA (2006). CKI, there's more than one: casein kinase I family members in Wnt and Hedgehog signaling.. Genes Dev.

[25] Li X, Yost HJ, Virshup DM, Seeling JM (2001). Protein phosphatase 2A and its B56 regulatory subunit inhibit Wnt signaling in Xenopus.. Embo J.

[26] Salic A, Lee E, Mayer L, Kirschner MW (2000). Control of beta-catenin stability: reconstitution of the cytoplasmic steps of the Wnt pathway in Xenopus egg extracts.. Molecular Cell.

[27] Yau TO, Chan CY, Chan KL, Lee MF, Wong CM (2005). HDPR1, a novel inhibitor of the WNT/beta-catenin signaling, is frequently downregulated in hepatocellular carcinoma: involvement of methylation-mediated gene silencing.. Oncogene.

[28] Jiang X, Tan J, Li J, Kivimae S, Yang X (2008). DACT3 is an epigenetic regulator of Wnt/beta-catenin signaling in colorectal cancer and is a therapeutic target of histone modifications.. Cancer Cell.

[29] Arce C, Perez-Plasencia C, Gonzalez-Fierro A, de la Cruz-Hernandez E, Revilla-Vazquez A (2006). A proof-of-principle study of epigenetic therapy added to neoadjuvant doxorubicin cyclophosphamide for locally advanced breast cancer.. PLoS ONE.

[30] Cong F, Schweizer L, Varmus H (2004). Casein kinase Iepsilon modulates the signaling specificities of dishevelled.. Mol Cell Biol.

